# Supplementary material for: The interactome of intact mitochondria by cross-linking mass spectrometry provides evidence for coexisting respiratory supercomplexes
Source: Mol Cell Proteomics. 2017 Dec 8;17(2):216–32. doi: 10.1074/mcp.RA117.000470 (PMC5795388; doi:10.1074/mcp.RA117.000470)
Supplement: Supplemental Data [file supp_RA117.000470_133922_0_supp_23978_fzffwf.zip › spectra_annotation/mito_spectra_annotation/36-1-58-1-5-1.pdf]

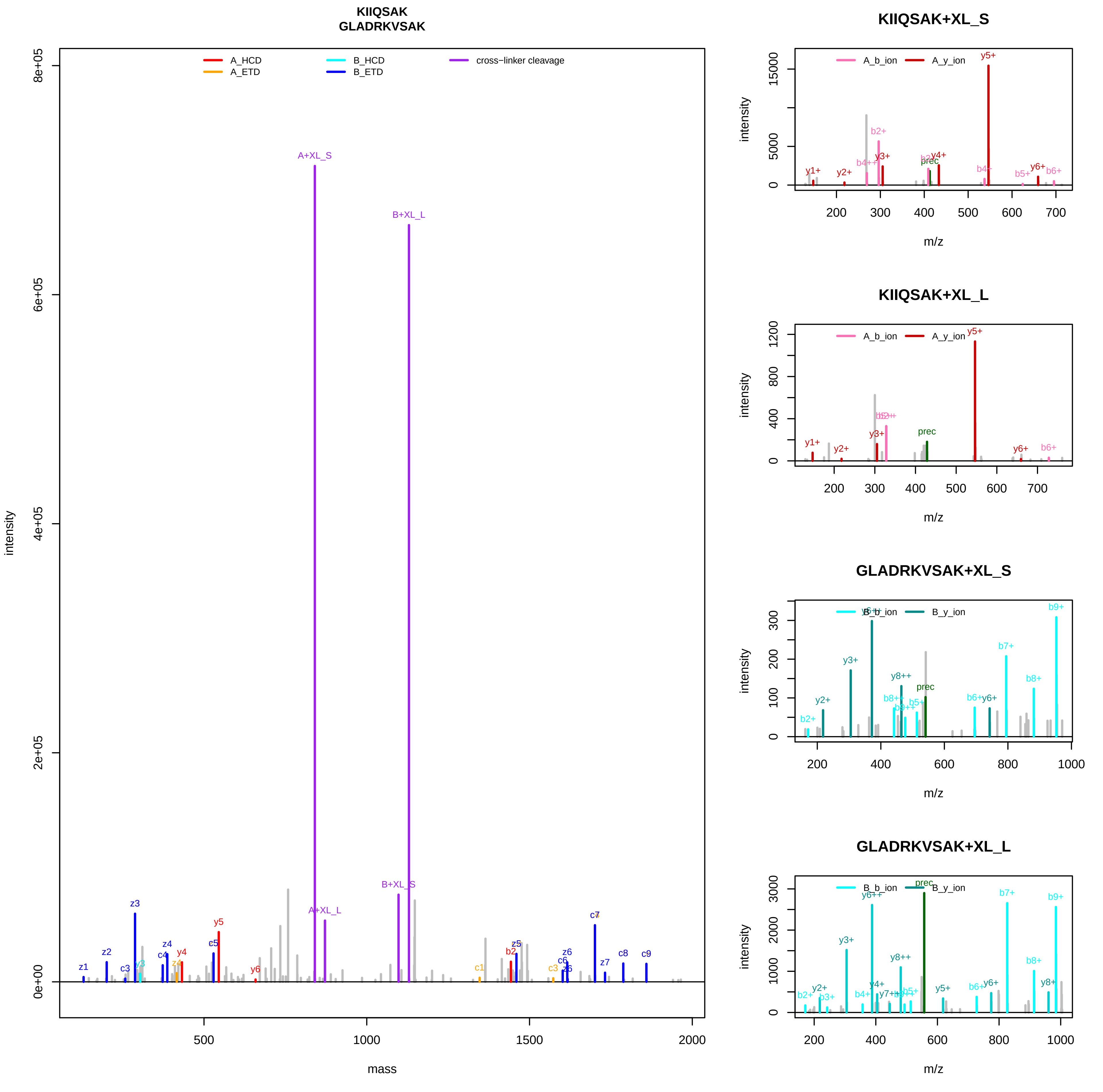
KIIQSAK
GLADRKVSAK
A_HCD
A_ETD
B_HCD
B_ETD
cross−linker cleavage
A+XL_S
B+XL_L
B+XL_S
A+XL_L
intensity
mass
0e+00
2e+05
4e+05
6e+05
8e+05
500
1000
1500
2000
z1
z2
c3
z3
y3
c4
z4
z4
y4
c5
y5
y6
c1
b2
z5
c3
c6
z6
z6
c7
z7
c8
c9
KIIQSAK+XL_S
A_b_ion
A_y_ion
y5+
b2+
b4++
y3+
b3+
y4+
prec
y1+
y2+
b4+
b5+
y6+
b6+
intensity
m/z
KIIQSAK+XL_L
GLADRKVSAK+XL_S
B_b_ion
B_y_ion
y3+
y8++
b8++
b9++
b5+
b6+
y6+
b7+
b8+
b9+
GLADRKVSAK+XL_L
y6++
y4+
y7++
y5+
y8+
b3+
